# Beyond enrolments: a systematic review exploring the factors affecting the retention of Aboriginal and Torres Strait Islander health students in the tertiary education system

**DOI:** 10.1186/s12939-019-1038-7

**Published:** 2019-09-02

**Authors:** Emma V. Taylor, Alex Lalovic, Sandra C. Thompson

**Affiliations:** Western Australian Centre for Rural Health, The University of Western Australia, 167 Fitzgerald Street, Geraldton, Western Australia 6530 Australia

**Keywords:** Indigenous students, Aboriginal and Torres Strait islander, Recruitment, Retention, Attrition, Academic success, Support, Health education, Higher education, Health workforce development

## Abstract

**Background:**

Indigenous Australians are under-represented in the health workforce, with large disparities between rates of Indigenous and non-Indigenous people in every health profession, including nurses, medical practitioners and all allied health professionals. Yet Indigenous people have long requested to have Indigenous practitioners involved in their health care, with this increasing the likelihood of culturally safe care. To address the shortage of Indigenous health professionals, it is important to not only recruit more Indigenous people into health courses, but also to support them throughout their studies so that they graduate as qualified health professionals.

The aim of this systematic literature review was two-fold: to identify the factors affecting the retention of Indigenous students across all tertiary health disciplines, and to identify strategies that support Indigenous students to remain with, and successfully complete, their studies.

**Methods:**

Eight electronic databases were systematically searched between July and September 2018. Articles were screened for inclusion using pre-defined criteria and assessed for quality using the Mixed Methods Assessment Tool and the Joanna Briggs Institute Checklist for Text and Opinion.

**Results:**

Twenty-six articles met the criteria for inclusion. Key factors reported by students as affecting retention were: family and peer support; competing obligations; academic preparation and prior educational experiences; access to the Indigenous Student Support Centre; financial hardship; and racism and discrimination. The most successful strategies implemented by nursing, health and medical science faculties to improve retention were multi-layered and included: culturally appropriate recruitment and selection processes; comprehensive orientation and pre-entry programs; building a supportive and enabling school culture; appointing Indigenous academics; embedding Indigenous content throughout the curriculum; developing mentoring and tutoring programs; flexible delivery of content; partnerships with the Indigenous Student Support Centre; providing social and financial support; and ‘leaving the university door open’ for students who leave before graduation to return.

**Conclusions:**

Universities have an important role to play in addressing inequities in the Indigenous health workforce. A suite of measures implemented concurrently to provide support, starting with recruitment and pre-entry preparation programs, then continuing throughout the student’s time at university, can enable talented Indigenous people to overcome adversities and graduate as health professionals.

## Background

The First Peoples of Australia have richly diverse and complex cultures which have existed for more than 50,000 years. However, colonisation disrupted traditional lifestyles, and discrimination and systemic disadvantage perpetrated upon this group of people means they have continued to experience poor outcomes in education and employment, and disproportionate levels of poor health [1–3]. The health disparity between Aboriginal and Torres Strait Islander peoples and non-Indigenous Australians across a wide range of indicators, including life expectancy, has been well documented for decades [1, 4, 5]. (The term ‘Indigenous Australians’ is hereafter used respectfully to refer to Australia’s Aboriginal and Torres Strait Islander peoples, and with full recognition of the tremendous diversity of the cultures and experiences of Australia’s First Peoples.)

There is a growing recognition of the multitude of factors that contribute to the poorer health status of Indigenous Australians [6]. Across the world, there is a strong connection between education and health outcomes, with mounting evidence in Australia and internationally, that successful participation by Indigenous people in higher education provides multiple benefits to the individual and the community [7–10]. Participation in a health sciences degree not only increases the educational attainment and earning potential of the individual with flow on effects to their family and community, it also increases the number of Indigenous people in the health workforce and consequently improves health outcomes for Indigenous people in the community [3, 11–16].

Australia urgently needs more Indigenous people within the health workforce. In 2015 they comprised only 1% of the registered health workforce, despite accounting for 3% of the Australian population and 4% of all hospital admissions [2]. Large disparities exist for every health profession, including nurses (in 2015, 1.1% of all employed nurses and midwives identified as Indigenous) and medical practitioners (0.5% of all employed medical practitioners identified as Indigenous) [17, 18]. Increased enrolment in and successful completion of health science degrees is therefore essential for developing an effective health workforce capable of meeting the needs of Australia’s First Peoples [19–21].

Many factors have contributed to the disproportionately small number of Indigenous people attending or completing tertiary education in Australia [10]. These factors range from long-standing historical policies that denied, restricted or segregated access to education [22, 23], to financial barriers [24] and current day perceptions that tertiary education, with its European traditions, is alienating, culturally unsafe or simply “not an option” [10, 25, 26]. Federal Government initiatives such as Closing the Gap and national curriculum reform, as well as intensive, sustained efforts by individual universities and faculties have led to a steady increase in the number of student enrolments [10]. Although there has been a 135% increase over the past decade in the number of Indigenous students enrolling in tertiary health degrees, population parity has not yet been reached, with Indigenous people currently comprising 2.1% of all commencing domestic health students [27]. Furthermore, improvements in recruitment have not been matched by improvements in retention, with course completions not increasing at the same rate as commencements. In fact, there is a widening gap between Indigenous and non-Indigenous students for health course completions, increasing from 11% in 2008 to 23% in 2017 [27]; in some courses, such as nursing, the gap can be much higher [28].

Graduation is only one measure of a good outcome from education and “success” is different for every student. Those who do not graduate may still use the learnings from their time at university in their careers and lives. However, to increase the number of qualified Indigenous health professionals necessitates graduation as a critical measure of success; this requires a focus on retention strategies to support students achieving this.

Despite numerous studies and reviews over the past three decades into the factors affecting success and retention for Indigenous Australians at university [23, 29–33], the majority of research “considers the university as a whole, with analysis of faculty attrition often neglected” ([33], p. 7). Consequently there is minimal evidence around educational strategies that achieve successful outcomes in supporting and retaining Indigenous health science students [26, 34, 35]. The purpose of this systematic literature review is two-fold: to identify factors affecting the retention of Indigenous students across all tertiary health disciplines from a *student* perspective, and to identify *strategies reported by health faculties* as effective in supporting those students and assisting retention. Wider promotion of these strategies may lead to them being more broadly adopted, increasing the number of Indigenous students graduating health science courses and becoming health professionals.

## Methodology

The review was conducted in accordance with the principles of the Preferred Reporting Items for Systematic Review and Meta-Analysis (PRISMA) statement [36], to meet standards for accurate and consistent reporting and with the aim of minimising methodological bias.

### Search strategy

**Fig. 1:**
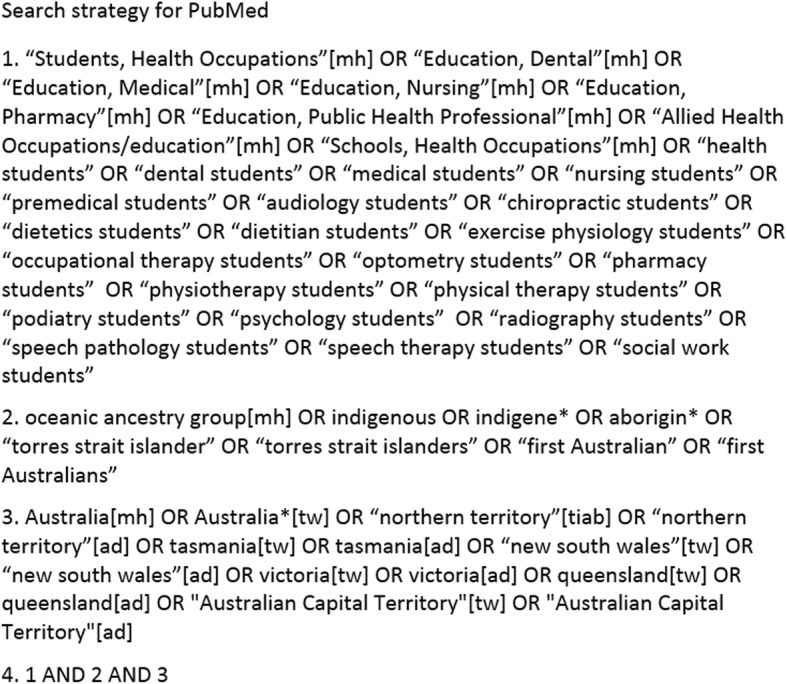
Electronic database search strategy example*. *Search terms varied slightly for each database

The search was conducted between July and September 2018, using database-specific search strings, across the following databases: PubMed, CINAHL, PsycInfo, Embase, Informit: Indigenous Collection, Informit: Health Collection, ERIC and Google Scholar. Key search words of ‘health students’, ‘Indigenous’ and ‘Australia’ were searched using a combination of subject headings and free text keywords (see Fig. 1). Back issues of the Australian Journal of Indigenous Education were hand searched, and reference lists of all retrieved articles (including other systematic reviews) were scanned manually.

### Screening process: inclusion and exclusion criteria

We included peer-reviewed empirical studies, peer-reviewed descriptive articles and grey literature reports that investigated the enablers or barriers to the retention of Aboriginal and/or Torres Strait Islander students within tertiary health courses, or which described or evaluated strategies to improve the retention of these students. Studies on all health courses were included. Two reviewers (EVT and AL) independently screened titles and abstracts of publications identified using the following predetermined exclusion criteria to determine eligibility for full text review: (i) language other than English; (ii) non-Indigenous students only; (iii) Indigenous population was patients rather than students; (iv) a tertiary course other than health; (v) focused on primary or secondary school students; or (vi) not based on findings from Australia.

The full texts were then independently reviewed with information extracted using a pre-determined data extraction form. Any disagreements regarding article eligibility were discussed and resolved by the two reviewers. A publication was excluded following full text examination if it: (i) included no findings or connection to retention or attrition related to Indigenous students (such as articles that just reported on positive or negative aspects of the course of study, without any connection to retention or attrition); (ii) reported on a tertiary course other than health or a qualification below Bachelor degree level; (iii) did not include a clearly defined Indigenous health student body; or (iv) was a literature review. Multiple papers from the same authors reporting on the same study population were included only if there were differences in the findings.

### Quality appraisal

The quality of the empirical studies (*n* = 14) and grey literature reports (*n* = 3) was assessed using the Mixed Methods Appraisal Tool (MMAT), Version 2018 [37]. The MMAT was selected as it is designed for systematic reviews that include qualitative, quantitative and mixed-methods studies, enabling the use of one tool for appraising the most common types of empirical studies [38] and it has been found to be efficient, reliable and has demonstrated content validity [39, 40]. After responding to two screening questions, each included study was rated in the appropriate category of criteria as either ‘yes’, ‘no’ or ‘unclear’. Of note, the tool does not address the quality of the reporting, but only the quality of the reported methods of the study. Two reviewers (EVT and AL) independently evaluated the articles.

Descriptive articles (*n* = 9) were assessed for quality by the same two authors using the Joanna Briggs Institute Checklist for Text and Opinion [41]. This tool consists of six criteria and allows for the appraisal of narrative text and expert opinion articles. Included articles were evaluated with respect to these criteria which include the established expertise of the author(s), the clarity and logic of the articulated argument, whether there was reference to existing literature and whether any incongruence with the literature was considered and defended [41]. Any score discrepancy was resolved through discussion.

After both quality appraisal tools had been completed and consensus reached, an assessment of ‘high’, ‘medium’ or ‘low’ quality was given to each article. It was decided at the outset that no study would be excluded on the basis of its quality assessment.

### Analysis

There were two components to the analysis of this review, which was conducted by two authors (EVT and AL) and then reviewed and refined by ST. Enablers, barriers, and recommendations relating to retention, as reported by current or former Indigenous students, were identified for each article. Factors affecting retention were then grouped into a matrix, which provided a logical framework to synthesise information. The matrix layout was based on one devised by Slatyer et al. [42] in their article looking at barriers and enablers to retention of Aboriginal Diploma of Nursing students and used with their permission. Finally, a count of identified factors provided a quantitative assessment of how frequently each factor was reported in the included literature.

Identified strategies or interventions to improve retention, as described or evaluated from the school or faculty perspective, were separated into individual components. Components were grouped chronologically to where they occurred during the students’ time at university and then mapped to a diagram. The frequency with which each strategy was reported was also assessed.

## Results

**Fig. 2:**
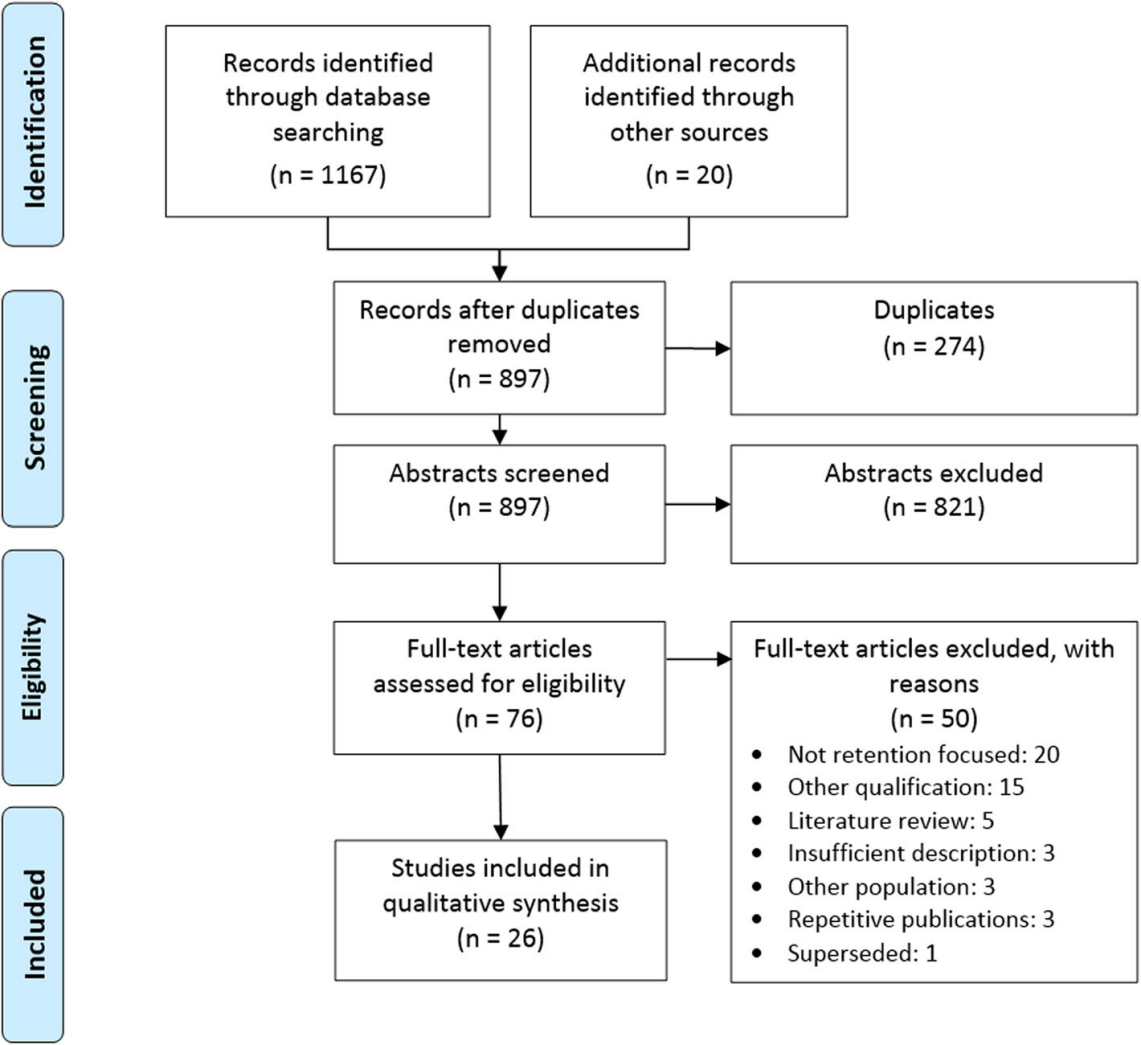
Search results and screening process based on PRISMA statement

We identified 26 articles that met our inclusion criteria. The results for each stage of our search and screening processes are shown in the PRISMA flow diagram (Fig. 2).

### Description of studies

Over half the articles included in this review were empirical (*n* = 14; 54%) (Table 1). The descriptive articles (*n* = 9; 35%) (Table 2) described strategies implemented to improve retention from the school or faculty perspective, but did not evaluate those strategies. Grey literature reports on the three main professions represented in the included articles (*n* = 3; 11%) are outlined in Table 3.

**Table 1:** Empirical studies

Author (Year)Location (University)	Methods	Study Population and Response Rate	Focus	Relevant Findings	Quality (MMAT)
Cameron et al. (2014) [43] Australia (multiple)	Qualitative Interviews	10 Indigenous psychologists. Response rate: unspecified. Purposive sampling and mass recruitment via e-mail to all members of the Australian Indigenous Psychologists Association.	Psychology Enablers and barriers for Indigenous students studying psychology.	Sources of support: family support, financial assistance, and Indigenous student support centres. Barriers: fear and anxiety about attending university, culture shock when relocating to a metropolitan area, lack of Indigenous staff and content, cultural insensitivity by staff and racism.	High
Chur-Hansen et al. (2008) [44] South Australia (University of Adelaide)	Qualitative Interviews	4 Indigenous students (1 medicine, 1 dentistry, 2 health sciences). Response rate: 100% of Indigenous first year students in the Faculty of Health Sciences.	Health Sciences Expectations and experiences of Indigenous health students at commencement and after 1 year.	Sources of support: wishing to make a difference for Indigenous health, the Indigenous student support centre and AIDA. Barriers: lack of knowledge about the course, lack of confidence, and family commitments.	High
Ellender et al. (2008) [45] Australia (multiple)	Quantitative Survey	12 Indigenous medical students who had deferred or withdrawn from their course. Response rate: 9% of withdrawn students. Purposive sampling, mass recruitment to 130 withdrawn students, advertisements in electronic newsletter.	Medicine Barriers experienced by 12 Indigenous medical students that caused them to withdraw.	Financial problems and relationship and/or family problems were the two most cited reasons for leaving. Other reasons for withdrawing included: high workload, fatigue, illness and cultural isolation, unclear expectations, and faculty staff. More support from the university may have encouraged respondents to continue.	Medium
Farrington et al. (2001) [46] New South Wales (University of Sydney)	Qualitative Interviews	26 Cadigal Program students from the following courses: physiotherapy, occupation and leisure studies, communication and speech disorders, nursing, medical radiation (unclear how many participated from each course). Response rate: unspecified.	Health Sciences Factors which influence participation, progression and retention of Indigenous students in full time health courses.	Sources of support: family, previous positive educational experiences, the Cadigal program and other Indigenous students. Factors that caused students to contemplate withdrawing: family and personal crises, financial difficulties and racism from non-Indigenous students. Strategy: the Cadigal program consisted of a two-week orientation program, the option of reduced load during first 2 years combined with the Aboriginal Health Science Support program, peer tutoring and access to facilities and resources.	Medium
Garvey et al. (2009) [47] New South Wales (University of Newcastle)	Qualitative Focus groups	16 Indigenous medical students. Response rate: 89% of Indigenous students enrolled in second or subsequent year.	Medicine Experiences of 16 Indigenous medical students and their perceptions of the factors influencing their progression.	Support provided by family, peers, senior Indigenous students and faculty staff was pivotal to students’ well-being and progression through training. Financial difficulties were cited as a reason to withdraw from studies. Other barriers that affected progression included: homesickness, personal and family issues, lack of confidence and racial discrimination.	High
Kippen et al. (2006) [20] Victoria (La Trobe University)	Qualitative Interviews, focus groups	16 participants (14 Indigenous (academics, public health students and key community stakeholders), 2 non-Indigenous public health academics). Response rate: unspecified. Purposive sampling.	Public Health Enablers and barriers affecting recruitment and retention of Indigenous Public Health students.	Family, positive role models and the Indigenous Student Support Centre were important sources of support. Barriers included: negative past educational experiences, family obligations, lack of Indigenous staff, cultural insensitivity by non-Indigenous staff and lack of formal articulation pathways from VET courses.	High
Mills et al. (2014) [48] Queensland (James Cook University)	Qualitative Interviews	11 Indigenous nursing students. Response rate: 92% of mentoring circle participants.	Nursing Describes the trial of a mentoring circle to support and retain Indigenous nursing students in a remote community.	Mentoring circle consisting of one or two mentors and 12 students met regularly over two semesters. Students formed a group identity and provided support to one another. As a group, students identified barriers affecting their ability to succeed at university and resolved those barriers through group discussions. Students identified skills required to succeed at university and developed those skills.	High
Schulz et al. (2018) [49] Queensland (Australian Catholic University)	Qualitative Focus groups	10 Indigenous midwifery students. Response rate: 77% of Indigenous students enrolled in the Away-from-Base Bachelor of Midwifery degree.	Nursing Evaluates two enhancements to a Midwifery course: appointment of an Indigenous Academic Liaison Midwife (IALM) and an additional clinical placement in a high-volume tertiary hospital.	Regular contact with the IALM helped students stay connected with and focussed on their study. Students respected the IALM as a culturally appropriate professional role model, who provided encouragement, cultural support and advocacy. The one week placement in a high-volume tertiary hospital was designed to minimise time students spent away from community. Students were supported by hospital staff and the IALM to ensure close supervision and culturally sensitive support.	High
Stuart et al. (2015) [50] Queensland (Not specified)	Qualitative Interviews, focus groups	5 Indigenous nursing students (former Indigenous Health Workers). Response rate: 100% of eligible students.	Nursing Enablers and barriers experienced by Indigenous Health Workers studying Bachelor of Nursing.	Recognition of prior learning and course exemptions alleviated workload stress and enabled students to complete their degree faster. Support from the Indigenous nurse academic and receiving financial support were cited as essential for students to remain at university. Other sources of support included: the Indigenous student support centre, a personal desire to make a difference and the support of family, workplace and other Indigenous students. All participants reported encountering racism during their course, which impacted on their desire to remain at university.	High
Usher et al. (2005) [51] Australia (multiple)	Qualitative Interviews	22 Indigenous nursing students. Response rate: unspecified. Purposive sampling.	Nursing Enablers and barriers experienced by Indigenous student nurses.	Adequate financial support was cited as critical for students to continue with their studies. Other important sources of support included: Indigenous Student Support Centres, support from non-Indigenous academics, support from family and other students and flexibility within the course. Challenges faced by students included racism, isolation and homesickness, family obligations and lack of adequate educational preparation.	High
West et al. (2013) [28] Australia (multiple)	Mixed methods Data analysis and interviews	Quantitative: 25 schools of nursing. Sample size: 65% of nursing schools. Interviews: 8 Indigenous nursing students, 13 nursing academics (5 Indigenous, 8 non-Indigenous). Response rate: unspecified. Purposive sampling.	Nursing Enrolment and completion rates for Indigenous student nurses across Australia. Student and staff perceptions of enablers to successful course completion.	National average completion rates are 36.3% for Indigenous nursing students and 64.6% for non-Indigenous nursing students (a difference of 28.3%). Individual student characteristics such as motivations for study, personal attributes (such as seeking support) and previous life and work experiences strongly affected their likelihood of successful completion. Family support, support from both Indigenous and non-Indigenous academics was also deemed critical for success.	High
West et al. (2016) [52] Queensland (Not specified)	Qualitative Interviews	8 final year Indigenous nursing students. Response rate: unspecified. Purposive sampling.	Nursing Indigenous nursing students’ perspectives enablers and barriers to their successful course completion.	Racism was identified as one of the biggest barriers to successful course completion. Previously identified barriers such as financial hardship and academic preparedness were no longer barriers due to students being more prepared to seek support. Students’ willingness to embrace support was identified as critical to successful course completion. Other personal attributes such as perseverance and a desire to make a difference for Indigenous health helped students to remain with their studies.	High
West et al. (2016) [53] Queensland (Not specified)	Qualitative Interviews	3 Indigenous midwifery students. Response rate: 100% of students who had provided continuity of care to Indigenous women.	Nursing Experiences of Indigenous midwifery students providing continuity of care to Indigenous women.	The relationships the students had with the Indigenous women, and the affirmation they received from those women and the wider community, gave students confidence and provided them with the motivation and resilience to continue with their studies.	High
Young et al. (2007) [54] South Australia (University of South Australia)	Quantitative Survey	33 current or former Indigenous students (17 nursing, 4 midwifery, 4 human movement, 1 occupational therapy, 3 physiotherapy, 1 podiatry, 1 naturopathy, 2 other). Response rate: 32% of Indigenous students who had been enrolled in a health sciences course between 2000 and 2005.	Health Sciences Investigates reasons for attrition of Indigenous health students and looks at support service usage.	Students’ reasons for withdrawing were varied and multifactorial. The most cited reason was difficulty balancing competing obligations. Other reasons for withdrawing included: literacy struggles, and lack of communication from the university leading to feelings of isolation and disengagement. Financial support and flexible delivery were identified as allowing students to remain with their studies when they otherwise would have withdrawn.	Medium

*MMAT* Mixed Methods Appraisal Tool

**Table 2:** Descriptive studies

Author (Year) Location (University)	Methods	Study Population	Focus	Relevant Findings	Quality (JBI T&O)
Best et al. (2014) [34] Queensland (University of Southern Queensland)	Qualitative Descriptive	None	Nursing Describes the Indigenous nursing support model: Helping hands.	The model is designed to individually mentor and support Indigenous nursing students. It consists of five steps: recruitment, orientation, retention, graduation and a supporting resource kit. Since implementing the model 80 Indigenous nurses and midwives have graduated from the program.	High
Fowler et al. (2018) [55] Western Australia (Edith Cowan University)	Qualitative Descriptive	None	Nursing Describes a conceptual framework and the action taken to support the recruitment, retention and academic success of Indigenous nursing students.	The Aboriginal and Torres Strait Islander Inclusivity Working Group (ATSIIWG) conceptual model consists of five elements: culturally responsive curriculum, cultural events, staff education, student involvement and community involvement.	High
Harris et al. (2012) [56] New South Wales (Charles Sturt University)	Qualitative Descriptive	Unspecified	Psychology Describes a model designed to address barriers for Indigenous psychology students.	Key elements of the model are: a whole-of-institution approach, embedding Indigenous content into the curriculum, partnership with the local area health service, mentoring and involvement of local elders and communities.	High
Hinton et al. (2010) [57] Northern Territory (Batchelor Institute of Indigenous Tertiary Education)	Qualitative Descriptive	Unspecified	Nursing Describes strategies implemented to improve Indigenous nursing students’ preparedness for entering the workforce.	Key strategies implemented were: holistic financial support (including accommodation, meals and free travel), a course timetable focused on reducing impact to family commitments while maximising clinical placement and nursing lab time, and a hospital-based mentoring program to support students while on clinical placement.	Low
Holliday et al. (2015) [58] New South Wales (University of Newcastle, University of New England)	Qualitative Descriptive	18 Indigenous pre-medical students.	Medicine Describes and evaluates the Miroma Bunbilla pre-entry to medicine program for Indigenous medical students.	The program is designed to strengthen the selection process for Indigenous medical students, ensure students have the required skills and improve retention. The program consists of a five day pre-entry intensive course. Students who completed the program in its first year had a 100% retention rate in their first year of study and gave positive feedback about the program.	High
Lawson et al. (2007) [59] Australia (University of Newcastle, University of Western Australia, James Cook University)	Qualitative Descriptive	Key representatives from three universities.	Medicine Describes the efforts of three medical schools to train and graduate Indigenous medical students.	Key strategies included: school-determined quotas for Indigenous students, alternative entry schemes, pre-medical preparation programs, flexible pathways into medicine and academic, social and personal support during the course. A rigorous selection process that assesses motivation, support structures and ability to balance study with other commitments helps to improve retention rates by selecting students who are more likely to successfully complete the course.	High
Meiklejohn et al. (2003) [60] Queensland (Queensland University of Technology)	Qualitative Descriptive	Unspecified	Nursing Describes strategies to increase the recruitment, retention and graduation of Indigenous nursing students.	Key strategies included: a streamlined and culturally-safe selection and admission process, a flexible study program including offering ‘leave of absence’ when required, a close relationship between the school and the Indigenous Student Support Centre, tutoring, personal contact with students, promoting peer networks and addressing racism.	Medium
Paul (2013) [61] Western Australia (University of Western Australia)	Qualitative Descriptive	None	Health Sciences Describes strategies to increase the recruitment and retention of Indigenous students studying medicine, dentistry and health sciences.	Key strategies included: alternative entry pathways for Indigenous students, individually tailored educational pathways (including a 1 year orientation course and a 5 week pre-medicine/pre-dentistry program), comprehensive and ongoing support for students throughout their degree (including access to resources, tutoring, assistance with applying for financial support and personal support), and integration of Indigenous content throughout curriculum.	High
Usher et al. (2005) [62] Queensland (James Cook University)	Qualitative Descriptive	None	Nursing Describes a Bachelor of Nursing course being delivered in the Torres Strait, incorporating specific strategies to improve recruitment and retention.	A satellite campus was established in a remote location with the aim of increasing recruitment of local Indigenous students and providing a more supportive environment that would lead to improved student retention. Key strategies included: employing local people, establishing a community consultative committee, a structured timetable with additional contact time to provide extra support, access to study resources, tutoring and establishing a mentoring program.	High

*JBI T&O* Joanna Briggs Institute Checklist for Text and Opinion

**Table 3:** Grey literature

Author (Year) Location	Methods	Study Population and Response Rate	Focus	Relevant Findings	Quality (MMAT)
Dudgeon et al. (2016) [63] Australia	Qualitative Consensus	None. Working party consensus with input from Indigenous stakeholders.	Psychology Guidelines for increasing the recruitment, retention and graduation of Indigenous psychology students.	Thirteen critical factors for increasing recruitment, retention and graduation: community partnerships, organisational leadership and enabling culture, mentors and role models, tutoring and academic support, scholarships and financial assistance, curriculum and pedagogy, community and family links, peer networks, IEC relationships, outreach and school visits, enabling and bridging programs, alternative entry, and quotas and designated places.	N/A
Indigenous Nursing Education Working Group (2002) [11] Australia	Quantitative Survey	22 schools of nursing. Response rate: 73% of nursing schools.	Nursing Framework to improve university-based Indigenous nursing education.	There is room for improvement regarding recruitment and retention strategies for Indigenous nursing students. Few schools of nursing have integrated Indigenous health into their core nursing curriculum. Makes 32 recommendations to improve Indigenous nursing education covering: recruitment, retention, curriculum development and implementation, advanced nursing practice and post-graduate education, articulation, partnerships and networks, and monitoring and accountability.	Medium
Medical Deans Australia and New Zealand et al. (2012) [64] Australia	Mixed methods Interviews, focus groups and audit.	19 medical schools. Response rate: 100% 133 Indigenous and non-Indigenous university staff, 142 medical students (44 Indigenous and 98 non-Indigenous). Response rate: unspecified. Purposive sampling.	Medicine Reviews the implementation of the Indigenous Health Curriculum Framework and the Healthy Futures Report.	Evidence that Indigenous medical students may have significantly higher withdrawal rates than non-Indigenous students. Quality and sustainability of recruitment and retention strategies for Indigenous medical students requires considerable attention within the majority of medical schools. Racism and discrimination remain a significant issue in the majority of Australian medical schools. Makes 10 recommendations to improve Indigenous medical education.	Medium

*N/A* Not applicable. This publication was not suitable for quality appraisal with the MMAT scoring system

Studies took place in all states and territories except Tasmania and the Australian Capital Territory. The largest number of studies took place in Queensland (*n* = 8) or were national in scope (*n* = 8). Two Queensland authors, West and Usher, were lead author on five of the included articles, all of which focused on issues affecting nursing students.

Half (*n* = 13) of the studies focused on issues affecting nursing and midwifery students [11, 28, 34, 48–53, 55, 57, 60, 62], five studies focused on medical students [45, 47, 58, 59, 64], three focused on psychology students [43, 56, 63], one study focused on public health students [20], and four studies reported on issues affecting Indigenous students across several health disciplines (courses included: dentistry, health science, human movement, medicine, nursing and midwifery, occupational therapy, physiotherapy and podiatry) [44, 46, 54, 61]. No articles reported specifically on the retention of dental students or any of the other allied health courses (such as social work or physiotherapy) apart from psychology.

Eighteen articles were assessed as good quality, six as medium quality, one was assessed as low quality [57] and one grey literature report was not suitable for quality appraisal using the MMAT because it was not an empirical study [63].

### Factors affecting retention

**Fig. 3:**
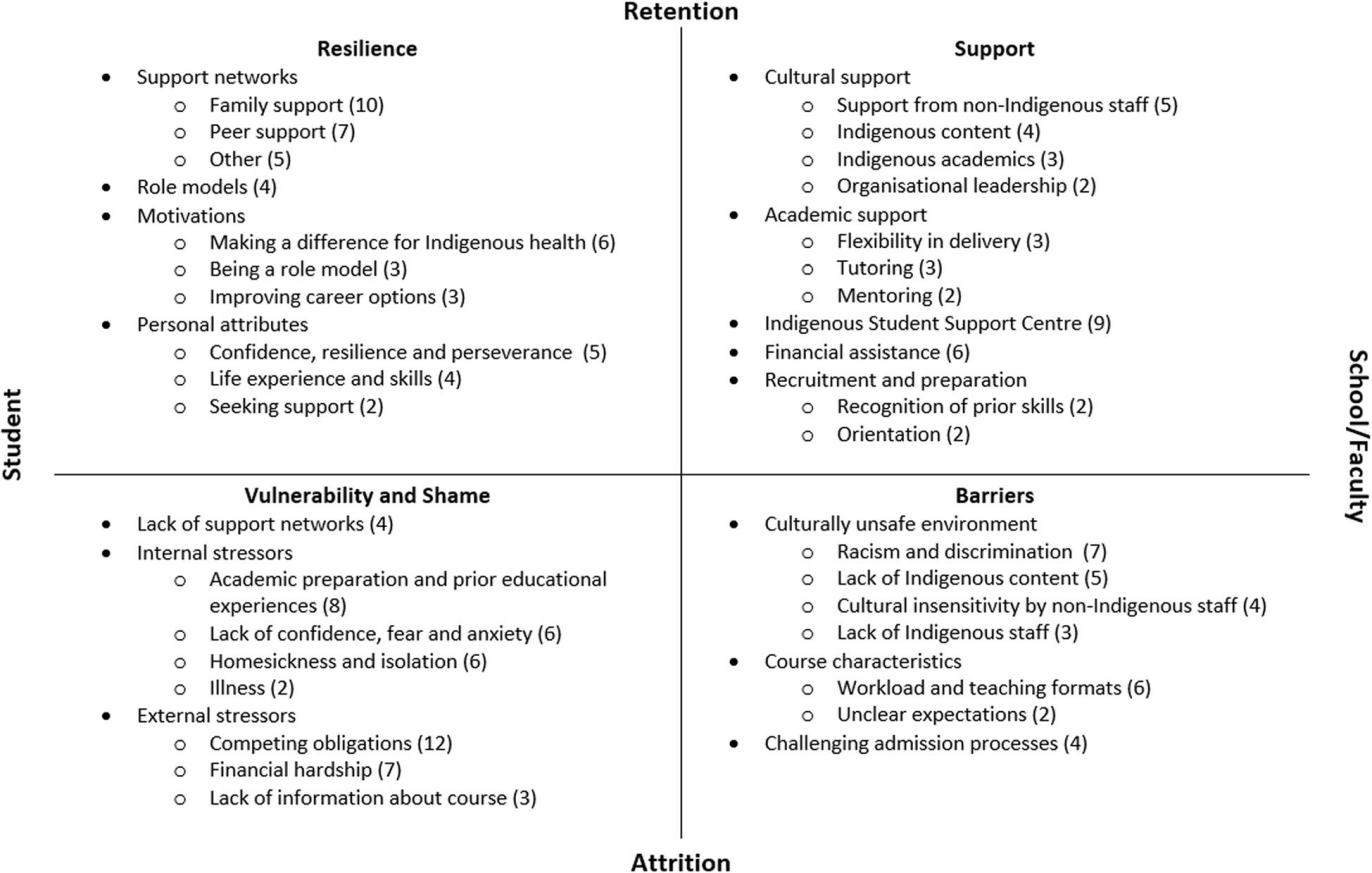
Factors affecting the retention of Aboriginal and Torres Strait Islander health students. Source: Adapted with permission from Slatyer et al. [42]. Numbers in round brackets refer to the number of articles identifying this factor

Factors reported by current or former Indigenous students as affecting retention were primarily identified from the empirical research articles. These factors have been represented via a matrix in Fig. 3. Rarely was one of these factors identified in isolation; generally it was the combination across several quadrants that resulted in students remaining or departing.

#### Student characteristics

A range of personal support networks were pivotal to students’ well-being and to their remaining at university. Family support was the most frequently mentioned enabler for retention [20, 28, 43, 46, 47, 50–53, 60] with family providing support, even when they hadn’t had educational opportunities themselves. Family support included emotional, financial and physical support, including when students were far away geographically, and it gave participants the confidence to go to university and encouraged them to remain there. Peer support from fellow Indigenous students, particularly those studying a health course, was also very important [28, 46, 48, 50–52, 60]. The value of support from workplaces and professional networks such as the Congress of Aboriginal and Torres Strait Islander Nurses and Midwives (CATSINaM) and the Australian Indigenous Doctors’ Association (AIDA) was also identified [44, 47, 50–52]. While not necessarily part of students’ support networks, senior Indigenous students and graduates of the course were inspiring and highly motivating role models for students, helping to engender confidence in their studies and encouraging them to stay at university [20, 46, 47, 52]. Conversely, where there was a lack of support or understanding from family or workplace, this contributed to students’ feelings of stress, isolation and loneliness [43, 45, 47, 50].

Internal motivations were a powerful driver for many students, with *wanting to make a difference for Indigenous health* the most frequently mentioned [28, 44, 45, 50, 52, 53]. Students also reported a desire to be a role model and inspire others within their family and community [50, 52, 53], and to improve their career options and “have a more influential role in [health] policy making” [45, 50, 53] as motivators for continuing with their studies.

A number of personal attributes were identified in the literature as having a protective effect; confidence, resilience and perseverance were all influential on course completion [46, 48, 51–53]. Some mature aged students reported feeling confidence as a result of their life experiences (previous employment, parenthood, positive educational experiences) and the skills they had acquired (such as study skills, communication, teamwork) [28, 46, 48, 51]. West et al. [28, 52] found that student nurses’ ability and willingness to seek out and then accept support was pivotal to successful course completion.

Having competing obligations was the most frequently mentioned barrier to remaining at university, reported by almost half of the included articles [20, 44–48, 50–52, 54, 57, 60]. Competing obligations included difficulties meeting family and community commitments, stress caused by family crises or illness, and difficulties balancing study, work and family. Financial hardship was mentioned by a quarter of articles as a reason for not continuing with study [20, 43, 45–47, 51, 60], and in some instances was exacerbated by feelings of shame for seeking financial assistance from others. Usher et al. [51] suggested that students’ financial burden had a cultural component as participants reported that they were expected to share money with extended family members, an expectation not usually experienced by non-Indigenous students.

#### School/faculty characteristics

Students described a range of cultural and academic support strategies implemented by schools and faculties that helped them continue their studies. Cultural support included supportive non-Indigenous academics and clinicians [28, 43, 50–52] who helped students to feel safe and comfortable at university. Support from Indigenous academics and clinicians was reported by students in three studies as “essential” to their remaining at university [28, 50, 52]. Culturally inclusive teaching and learning practices with consideration of different student learning styles, as well as embedding Indigenous content into the curriculum, was reported as assisting retention by students in four studies [28, 51, 52, 63].

Racism and discrimination were barriers reported in over a quarter of included studies [43, 46, 47, 50–52, 64], with West et al. [52] describing racism and discrimination as “one of the most pervasive and debilitating barriers to successful course completion” (p. 353). Examples ranged from questioning the student’s Indigeneity, to discrimination and a lack of acceptance by students and staff because the student was viewed as receiving ‘special treatment’.

Schools or faculties could support students academically by being flexible in their delivery of course content and allowing students to move between study modes (internal/external, full-time/part-time) [28, 51, 54]. This was reported as enabling individuals to modify their mode of study according to personal needs, allowing them to “hang-in there” ([54], p. 40). Access to tutors [52, 54, 63] and mentoring [63, 64] helped create a safe, supportive space for students.

The Indigenous Student Support Centre was reported by participants in a third of articles [20, 28, 43, 44, 46, 47, 50–52] as providing academic, social, emotional and cultural support, including tutoring, and advice on finances, scholarships and accommodation. Receiving financial assistance such as a scholarship, cadetship, or ABSTUDY (special government benefits for Indigenous students in an approved course of study), helped students to remain with their studies, especially during clinical placements when they generally had to take leave from their paid employment [43, 50–52, 54, 63].

During the recruitment and preparation stages, students reported that recognition of their existing skills and prior learning helped them feel “empowered” and valued; when course exemptions were granted, this contributed to improved retention by helping students complete their degree faster and reducing financial pressures [50, 63]. Comprehensive orientation programs helped students to understand school or faculty expectations, meet other students and feel accepted and welcomed by staff [46, 51]. Conversely, a challenging admission process, such as a lack of formal pathways from vocational education and training (VET) courses that articulated with university studies, or difficulties applying for recognition of prior learning (particularly for Indigenous Health Workers), contributed to students feeling stressed, disempowered and undervalued [20, 50, 60, 64].

### Strategies to improve retention

**Fig. 4:**
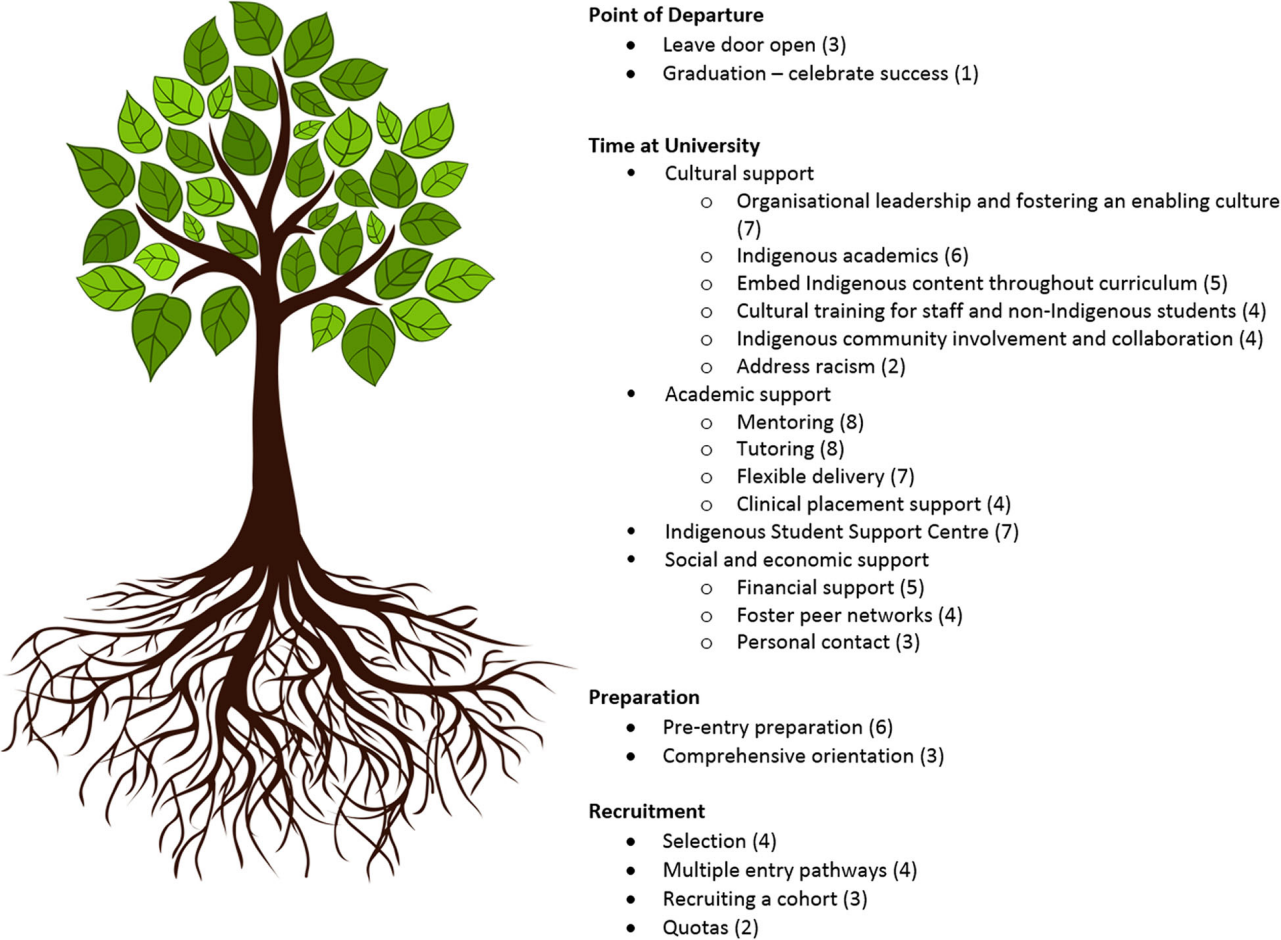
Strategies for growing strong students. Numbers in round brackets refer to the number of articles identifying this strategy

Descriptions of strategies or programs implemented by health schools or faculties to improve retention were primarily identified from the descriptive articles, and are represented chronologically in Fig. 4.

#### Recruitment

Multiple articles, predominantly those looking at strategies implemented in medical schools, claimed that retention starts with appropriate selection [58–60, 64]. Medical schools aimed to select students who would successfully complete the course by looking at applicants’ motivations, support structures and ability to balance study with other commitments. Lawson et al. [59], in their discussion of the strategies adopted by the three leading universities regarding Indigenous student enrolment in medicine, reported a strong link between selection and retention, although they acknowledged the need to achieve a balance between achieving a high completion rate and giving “higher-risk” students a chance. Two articles emphasised the importance of selecting students through a culturally appropriate interview process [60, 64]. The importance of offering multiple pathways into medicine was also stressed [58, 59, 61, 64].

Three studies identified the importance of a cohort effect with Indigenous students enabling peer learning and support [46, 58, 60], while others identified the importance of course quotas with specific places available for Indigenous students [11, 59]. Being with other Indigenous students directly benefitted students’ academic success and likelihood of retention, reflecting the moral and academic support students can provide to one another. For example, Meiklejohn et al. [60] concluded (p. 7):

"A critical mass of eight students has been identified as the minimum number of enrolees at any one time likely to result in successful completion of studies. Numbers of less than eight may leave students feeling isolated, and without study partners, encouragement and peer support."

#### Preparation

Articles described pre-entry preparation programs and comprehensive orientation programs as an effective way to inform students about the support available to them at university, as well as communicating university expectations and managing student expectations. Descriptions of pre-entry preparation programs were relatively common [54, 56, 58, 59, 61, 64], although predominately employed by medical schools. Courses described as “pre-entry” varied substantially in terms of the aims, content covered and duration, with examples ranging from 5 days to 1 year. Holliday et al. [58] described a comprehensive 5 day pre-entry to medicine intensive course which demonstrated positive outcomes, including 100% retention rate during first year for participants of the program, and “overwhelmingly” positive feedback from students. Comprehensive orientation programs were less commonly described [34, 46, 58] and ranged from 1 day to 2 weeks in duration. As one component of the Indigenous Nursing Support (INS) model: Helping Hands, Best et al. [34] described an orientation tool developed for Indigenous student nurses, which included the ‘Dandiiri’ orientation breakfast, a special enrolments area and formally allocated staff support.

#### Time at university

Providing cultural support for Indigenous students was recognised as needing to come “from the top”, with over a quarter of the articles reporting strong organisational leadership and a deliberate attempt to foster an enabling culture within their schools or faculties [28, 34, 55–57, 59, 64]. Leadership at the institutional and faculty or school level had flow-on effects within the organisational hierarchy, creating an enabling culture which incorporated Indigenous knowledge and sought to support and respect the Indigenous identity of students. Fostering cultural safety required commitment from every academic rather than relying on one or two motivated staff or only Indigenous staff.

Schools and faculties also demonstrated a commitment to cultural safety by implementing cultural awareness training for non-Indigenous academics and students [11, 55, 56, 64]. However, a review co-authored by the Medical Deans Australia and New Zealand (Medical Deans) and AIDA [64] found that these programs were generally targeted towards students rather than staff, that attendance was voluntary and they were often viewed as being “tokenistic and counterproductive” (p. 35). Specific strategies to address racism were only reported in two articles [59, 60]. Meiklejohn et al. [60] briefly described a number of implemented strategies to address racism including offering counselling and ongoing support to the victims, an address by the Head of School to the student body and disciplinary action taken against the perpetrators, however, there was no indication of how successful these strategies were. Stuart et al. [50] recommended implementing a formal complaint process (including an anonymous hotline number) and employing Indigenous counsellors to give advice to staff and students as a response to the racism commonly experienced by the participants in their study.

Indigenous academics supported students culturally and academically [28, 34, 44, 49, 50, 62] with regular contact helping students stay connected and focused on their studies. Schulz et al. [49] describes how the new Indigenous Academic Liaison Midwife (IALM) was respected by students “as a culturally appropriate professional role model” who advocated for students with the other academics, with one student stating “if it wasn’t for [the IALM] I wouldn’t have made it this far” (p. 62). Also supporting students culturally and academically was the inclusion of Indigenous content in the core health curriculum [34, 55, 56, 61, 64], which made the curriculum more relevant for Indigenous students (increasing the likelihood of retention) and reinforced the belief that Indigenous health issues were worthy of discussion [34, 56]. Two articles described how Indigenous content was developed in consultation with local Indigenous people to ensure that materials were respectful and safe [55, 64]. None of the articles described the exact nature of the Indigenous content, only that it “foregrounds Indigenous world views, cultures and experiences” ([56] p. 131), although an audit of the curriculum of all Australian medical schools by Medical Deans et al. [64] found that Indigenous health content covered a wide range of topics including “rural Indigenous health, cultural awareness and cultural implications for health, Indigenous history, communication, clinical presentations of disease, population health and social determinants of health” (p. 12). The amount of Indigenous content incorporated into the curriculum varied amongst the five articles and ranged from one core undergraduate unit [34, 55] to a combination of core units and electives throughout the course [56, 61], to Indigenous health not being taught discretely but as “integrated components of broader subject areas” ([64] p. 12). Schools and faculties reported involving the local community through service programs, clinical placements at the local Aboriginal Medical Service, and talks from Elders and local community members [34, 55, 56, 62].

The two most commonly described strategies to provide academic support were targeted mentoring programs [11, 34, 48, 57–59, 62, 64] and tutoring [11, 34, 46, 58–62]. Mills et al. [48] described the implementation of mentoring circles (a form of group mentoring) as a successful way to support Indigenous nursing students during their studies by empowering them to develop the skills they identified as needing to complete their studies. The review by Medical Deans et al. [64] described a mentoring program where social and academic support and professional guidance was provided by experienced (non-Indigenous) clinicians, with some students regarding the mentors as “akin to Elders” (p. 33). Four articles briefly described strategies to support nursing students during clinical placement [34, 49, 53, 57]. Best et al. [34] mentions the “Coolamon clinical school” as a service that provides “support with clinical nursing placements for all Indigenous nursing students” although the exact nature of that support was not specified (p. 64).

Flexibility in the delivery of course content was another way to support students academically [11, 46, 49, 56, 57, 60, 62]. Some schools or faculties aimed to reduce the travel burden to students by delivering the course in block mode, and Hinton et al. [57] described a Bachelor of Nursing timetable that specifically focused on reducing any impact to family commitments while maximising clinical placement and lab time. Both Meiklejohn et al. [60] and Farrington et al. [46] described how students could choose to reduce their study load, while concurrently completing tutoring or an Aboriginal health science support program, with participants in Farrington’s study reflecting that they would not have coped with a full load.

The two most commonly described strategies to provide social and economic support were financial support [11, 57, 59–61] and fostering peer networks [34, 48, 55, 60]. Descriptions of financial support included: assistance applying for scholarships and cadetships; access to class sets of text books, laptops, diagnostic kits; and funding to attend conferences or a travel allowance during clinical placements. Schools supported and encouraged Indigenous students to network by organising social events such as breakfasts and morning teas as an informal way for students to meet. No articles reported on strategies to encourage networking between Indigenous and non-Indigenous students, although Harris et al. [56] described a planned buddy system that would encourage Indigenous and non-Indigenous students to work together to “provide first-hand experience of reconciliation in action, and important exposure to perspective taking” (p. 133). A small number of articles described ‘personal contact’ as a strategy for retaining students [34, 60, 64], with feedback from students in one study indicating that this was “critical to success” ([64], p. 33).

#### Point of departure

Only Best et al. [34] mentioned the importance of celebrating graduation, with the inclusion of a graduation tool within the INS model: Helping Hands to help nursing students celebrate their success. However, Harris et al. [56] described how progress was recognised throughout a Graduate Diploma in Psychology program, by providing a certificate after the first year was completed and a diploma at the end of second year. This had the added advantage of providing “exit points” throughout the qualification. Harris et al. [56] explains (p. 133):

“The reality is that not all students will make it through a 3- or 4-year degree. For many students who are the first generation of their family to attend university, a certificate or diploma recognising what they have done is an accomplishment. It provides a way to exit without losing face, and hopefully encourages them to come back, or, to pursue some other tertiary qualification. It also signals to an employer that the student has attempted tertiary training in psychology.”

The importance of “leaving the university door open” for students who left before graduation, was also mentioned in two other articles [59, 60]. Strategies to prevent students withdrawing unnecessarily included offering a leave of absence when a student’s progress stalled due to outside factors, and the option of transferring to a less onerous course of study for a year if the student is struggling academically.

## Discussion

This paper has explored issues associated with retaining Indigenous Australian students in tertiary health courses and identified 26 articles reporting factors affecting retention or that described strategies implemented by schools or faculties to improve retention. Despite the pressing need for Indigenous health professionals, there were relatively few published strategies to improve the retention of health students, minimal documented evaluation of these strategies and no intervention trials, with limited evidence about which retention strategies are most effective. Far more has been written about the factors affecting retention, possibly reflecting the ease with which challenges can be described and the difficulties with achieving robust evidence into what makes a difference in improving outcomes.

Key factors reported by students as affecting retention were: family and peer support; competing obligations; academic preparation and prior educational experiences; access to the Indigenous Student Support Centre; financial hardship; and racism and discrimination. This accords with the findings of previous national and international reviews on Indigenous nurses [19, 26, 65], as well as findings about the retention of Indigenous health students in vocational education [42, 66, 67] and Indigenous Australians in other fields of study [68–70].

The majority of retention strategies reported in the literature were focussed solely on the students’ time at university, with recruitment and retention often tackled as separate issues. However, we found that recruitment and retention are linked, with persuasive reports that the process of selection and preparation for university is critical to a student’s retention [58, 59, 64]. Using the metaphor of a tree, if a tree doesn’t have strong roots, it may still grow, but the trunk won’t be as strong to withstand winds, and the tree won’t produce as many leaves, flowers or fruits. In the same way, if the groundwork isn’t done with the students – if they aren’t informed about the support available to them at university or the expectations of the course prior to starting, if they don’t acquire the skills they might need, then they are likely to struggle at university, especially if other challenges occur in their life. We chose to represent the strategies implemented within universities for retaining students using the tree analogy, with the strategies grouped chronologically based on where they supported a student during their study timeline (see Fig. 4).

Despite racism and discrimination being reported as major barriers to retention by students and academics across multiple studies [43, 46, 47, 50–52, 64], few schools or faculties mentioned racism as an issue within their institution and only two articles gave specific examples of strategies to address racism [59, 60]. While broader strategies such as organisational leadership and fostering an enabling culture, and cultural training for staff and students, are clearly aimed at creating a safe place for Indigenous students, there seemed to be a lack of willingness to acknowledge either institutional or interpersonal racism as a problem. This may be because schools and faculties lacked insight, because they feared repercussions for admitting that racism existed in their faculty, or because they felt racism was adequately addressed by university-wide policies. Anecdotally there seems to be a lot of uncertainty about appropriate strategies to address racism within schools and faculties. A report by Rodgers-Falk et al. [70] exploring initiatives in the higher education sector to increase the number of Indigenous Australian law graduates, states that “we cannot develop [Aboriginal and Torres Strait Islander] inclusiveness effectively without being able to identify racism, understand its gravity, and being prepared to deal with it. Schools need to have a racism strategy that includes - responding, reprimanding, and policy implementation” (p. 3). The Universities Australia ‘National best practice framework for Indigenous cultural competency in Australian universities’ may be a useful guide, as it is designed to provide universities with the tools required to create culturally supportive environments for Aboriginal and Torres Strait Islander students and staff [71].

One notable gap in the literature was any investigation of Indigenous students’ experiences while on clinical placement and whether their experiences had any effect on their decision to remain with or depart from their studies. In addition, there was a lack of reported strategies to support Indigenous students while on clinical placement, with only four nursing or midwifery articles mentioning clinical placement support [34, 49, 53, 57]. While this accords with the findings of a previous review on Indigenous nurses [26], it is troubling because clinical placements are a core component of many health science degrees as well as preparing students for the workforce. As Milne et al. [26] states, “awareness of students’ experience in this context is essential to their academic success” (p. 392).

Effective, long-term strategies to grow the Indigenous health workforce cannot start at university. Skills and knowledge learned at school are critical elements of the health workforce development pipeline (and therefore workforce development policies and university recruitment strategies) [72, 73]. Although out of scope of the current review, there needs to be a greater focus on increasing the number of Indigenous students completing high school, encouragement of Indigenous students to study science in high school, and support for students during their transition from high school to university [64, 72]. In addition, there needs to be a focus on articulation and increasing the pathways and support available for students wishing to progress from VET health programs into graduate entry programs.

### Limitations

Systematic reviews are inevitably limited by the quality and quantity of research available for inclusion. The literature was primarily descriptive in nature, with only 14 empirical studies identified, and while most studies made recommendations or described implemented strategies, none had tested an intervention aimed at improving retention. The lack of rigorous evaluations measuring the effectiveness of retention strategies for Indigenous health students has previously been identified [34, 72]. This study was also limited by the methodological quality of some articles. However, the limitations of included studies have been acknowledged and communicated through quality scores, allowing readers to take this into consideration. Furthermore, many of the included studies had small sample sizes (only one study had more than 33 participants) or gave limited data on the demographics of the population (8 studies only provided the students’ field of study), which limits the generalizability of the findings. Over a third of articles (38%) are more than a decade old, and pre-date the Australian Government Closing the Gap initiative to reduce inequalities in Indigenous life expectancy, mortality, education and employment [74]. Of greater concern is the observation that there has been no apparent increase in the rate of publication in this field (unlike the exponential increase observed within Indigenous health research [75]), suggesting that this is not a growing research priority. These limitations in the literature, with reports that are observational, descriptive and cross-sectional in nature, underscore the urgent need for robust evaluation and research in this area.

### Implications

Universities have an important role to play in addressing inequities in the Indigenous health workforce. It is hoped that findings from this review will be used by schools and faculties to inform the development of support strategies and programs that incorporate:

- The whole of student life; starting with recruitment and selection, continuing through pre-entry preparation and orientation, and including cultural, academic, social and economic support while at university, as well as considering point of departure.
- Opportunities for students to meet and connect with fellow Indigenous students during orientation and throughout their studies, whether through formal mentoring programs, student networks or informal social gatherings.
- Flexibility in delivery.
- Engagement with and input from local Indigenous communities and health services.
- Evaluation and assessment of efficacy, with the publication of results where possible, so that a body of ‘best practice’ evidence can be established.

In addition, schools and faculties need to adopt and communicate policies, guidelines and actions that address racism. The Universities Australia ‘National best practice framework for Indigenous cultural competency in Australian universities’ outlines guiding principles and specific recommendations with the aim of making Australian universities places where Indigenous students can “thrive and feel at home” ([71], p.7).

Government also has a role in delivering and supporting effective schemes that provides incentives and financial support for Indigenous students, and proactive policies that encourage universities to provide the additional supports needed by Indigenous students.

Clinical placements are a core component of many health science degrees. Despite this, we found no investigation of Indigenous students’ experiences while on clinical placement and few reported strategies to support Indigenous students while on clinical placement. This highlights the need for further research in this area, with collaborations between academics and clinicians and between universities and health services, to establish the factors affecting success for Indigenous students while on placement and how best to support students, while at the same time preparing them for the workforce.

## Conclusions

The enablers and barriers to Aboriginal and Torres Strait Islander student retention have been researched for 30 years. This systematic review, focussed on health science courses, reiterates the findings from a multitude of previous studies that the key factors affecting retention (as reported by students) are: family and peer support; competing obligations; academic preparation and prior educational experiences; access to the Indigenous Student Support Centre; financial hardship; and racism and discrimination. Historically, there has been little written about practical strategies to support Indigenous students. This review found that the most successful strategies implemented by nursing, health and medical science faculties to improve retention were multi-layered and started before the student commenced at university. Specific strategies included: culturally appropriate recruitment and selection processes; comprehensive orientation and pre-entry programs; building a supportive and enabling school culture; appointing Indigenous academics; developing mentoring and tutoring programs; flexible delivery of content; partnerships with the Indigenous Student Support Centre; providing social and financial support; and ‘leaving the university door open’ for students who leave before graduation to return.

The gap in the literature that became apparent is empirical research that measures the efficacy of strategies to improve retention. While commendable to see schools and faculties describing their many efforts, programs and strategies to improve retention, the evidence that supports the efficacy of these approaches is limited. Although trial designs are unrealistic, programs and strategies need to be evaluated with pre- and post-implementation measures, and assessed using both qualitative and quantitative data/research. As stated by Best et al. [34] in 2014, “The underlying problem is not one of policy direction, but one of policy implementation. Little current literature provides discussion about best practice in achieving successful outcomes in graduating Indigenous nurses” (p. 61). And while it is tempting to look for a “magic bullet” in terms of implementing what seems most effective, it seems certain that a suite of measures implemented concurrently to provide support across multiple domains will most enable talented Indigenous people to overcome adversities and graduate as health professionals. The skills learnt while achieving this milestone will also be valuable as they enter the workforce, where retention of Indigenous health professionals remains an important concern [76].

## Data Availability

Not applicable.

## Abbreviations

AIDA: Australian Indigenous Doctors’ Association
CATSINaM: Congress of Aboriginal and Torres Strait Islander Nurses and Midwives
IALM: Indigenous Academic Liaison Midwife
INS: Indigenous Nursing Support
JBI T&O: Joanna Briggs Institute Checklist for Text and Opinion
Medical Deans: Medical Deans Australia and New Zealand
MMAT: Mixed Methods Appraisal Tool
PRISMA: Preferred Reporting Items for Systematic Review and Meta-Analysis
VET: Vocational education and training
